# Model-Measurement Comparisons for Surfactant-Containing Aerosol Droplets

**DOI:** 10.1021/acsearthspacechem.4c00199

**Published:** 2024-10-22

**Authors:** Alison Bain, Nønne L. Prisle, Bryan R. Bzdek

**Affiliations:** † School of Chemistry, University of Bristol, Cantock’s Close, Bristol BS8 1TS, U.K.; ‡ Department of Chemistry, Oregon State University, 2100 SW Monroe Ave, Corvallis, Oregon 97331, United States; § Center for Atmospheric Research, University of Oulu, Oulu 90014, Finland

**Keywords:** surface tension, surface-area-to-volume ratio, bulk depletion, partitioning model, hygroscopic growth, cloud droplet activation

## Abstract

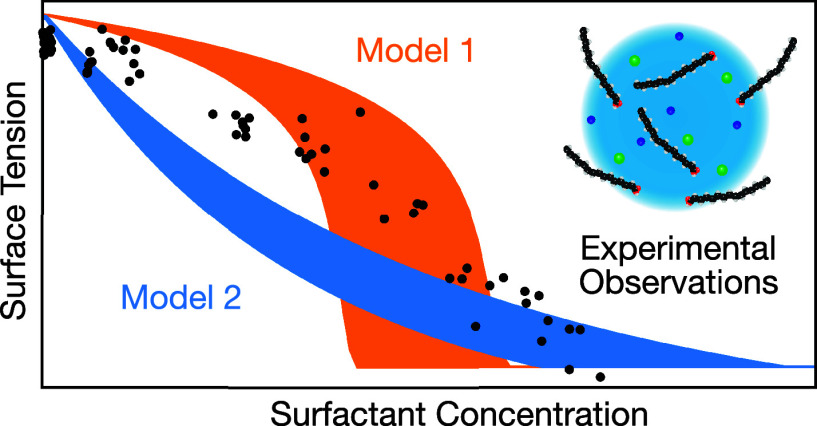

Surfactants are important components of atmospheric aerosols, potentially impacting their hygroscopic growth and eventual activation into cloud droplets. By adsorbing at the air–water interface, surfactants lower the surface tension of aqueous systems. However, in microscopic aerosol droplets, the bulk surfactant concentration can become depleted because of the droplets’ high surface-area-to-volume ratio, reducing the bulk surfactant concentration at equilibrium and increasing droplet surface tension. Partitioning models have been developed to account for the concentration- and size-dependencies of surface tension, but these models have rarely been assessed against experimentally measured droplet surface tensions. Here, we directly compare surface tension predictions made using a simple kinetic partitioning model and a thermodynamic monolayer partitioning model against experimentally measured picoliter droplet surface tensions for 12 surfactant–cosolute systems. Surface tension predictions were also made across 8 orders of magnitude in droplet radius. The largest differences between model predictions were associated with the predicted onset of bulk depletion. The quality of the isotherm or parametrization fit to the macroscopic data most strongly influenced a model’s ability to accurately predict droplet surface tension. These results highlight the importance of validating partitioning models against droplet surface tension measurements in size ranges where bulk depletion is expected to occur and motivate collection of high-quality macroscopic surface tension data sets that serve as model inputs. The results also validate both models’ abilities to predict aerosol surface tension across size and composition, which will facilitate their eventual incorporation into cloud parcel models to explore the impact of surface tension assumptions on cloud droplet number concentration.

## Introduction

Atmospheric aerosols affect climate by directly scattering solar radiation (the direct aerosol effect) and by impacting cloud microphysics (the indirect aerosol effect).^1^ Surface active molecules are an important component of aerosol chemical composition because these molecules can partition to the droplet surface and in principle lower surface tension.^2−4^ A reduction in surface tension due to surface active organics has been suggested to explain discrepancies between cloud condensation nuclei (CCN) concentrations observed in field studies and those predicted using Köhler theory.^5−8^ A reduced surface tension lowers the barrier to cloud droplet activation, resulting in more aerosol droplets activating at a given atmospheric supersaturation than would be expected assuming a surface tension of pure water, ultimately altering cloud properties.^9^ Beyond atmospheric implications, surface tension is a necessary property to understand the hygroscopic growth of respiratory and other aerosols,^10,11^ accelerated reaction rates and unique chemistry at the surfaces of droplets,^12−17^ and the structure and morphology of spray dried particles.^18,19^ However, predicting surfactant partitioning is complex, depending on both chemical composition and droplet size.^20^ In large surface-area-to-volume (SA-V) ratio systems like microdroplets, a greater proportion of the total molecules are adsorbed to the surface at equilibrium compared to a macroscopic solution with the same total composition,^21^ leading to depletion of the droplet’s bulk concentration and potentially an increase in the droplet’s surface tension.^22^

Several partitioning models have been developed using different frameworks to predict aerosol droplet surface tension.^7,22−31^ Previous studies have directly compared multiple partitioning models for Nordic Aquatic Fulvic Acid and NaCl mixtures at various organic mass fractions^22^ and aqueous salt solutions (NaCl or (NH_4_)_2_SO_4_) containing succinic acid, sodium dodecyl sulfate, Nordic Aquatic Fulvic Acid, or Pollenkitts for a range of dry diameters and organic mass fractions.^26^ Vepsäläinen and coworkers performed two of the most extensive model comparisons to date, comparing the supersaturation required for 50 nm dry diameter NaCl/sodium myristate and NaCl/malonic acid aerosol at different organic mass fractions to activate into cloud droplets across five partitioning models.^32,33^ In all these studies, the model predictions were compared to critical supersaturations obtained using a CCN counter.^34^ However, such an approach provides only a single data point to test against complex models that generate rich data sets across droplet size and composition, and Köhler calculations show that the SA-V ratio of droplets at activation plays a large role in how well different partitioning models predict critical supersaturations.^22,32,33^ Critical supersaturation is influenced by mutually dependent parameters, including surface tension reduction and size-dependent surfactant partitioning, making CCN measurements insufficient to fully assess the appropriateness of any given model to describe size-dependent surface tension. Comparisons of partitioning models against experimentally measured aerosol droplet surface tensions are complementary to this approach and permit assessment of the model across a much wider range of parameter space, leading to a more robust model evaluation.

The lack of detailed comparisons to model-predicted aerosol droplet surface tensions arises from the lack of experimental reference measurements. A range of techniques have been developed to measure the surface tension of picolitre volume droplets suspended in air,^35−39^ but few have been used to investigate concentration- and size-dependent partitioning of strong surfactants.^40^ Experimental observations of bulk depletion in aerosol droplets have only been compared to predictions for the surface tension from the full, thermodynamically consistent Gibbs partitioning model^41^ and the Monolayer Partitioning Model.^41,42^ The Gibbs model was found to dramatically overpredict the measured droplet surface tensions, in part due to the poor constraint on required activity coefficients and no constraint on the amount of partitioning that can take place, but the Monolayer Model, which explicitly determines the partitioning of each droplet component—water, surfactant, and cosolute—showed good agreement with experimental droplet measurements.^41,42^ The qualitative agreement of the Monolayer Model with experimentally determined droplet surface tensions and its ability to predict the surface and bulk concentrations of all species in a droplet simultaneously make it a good candidate for accurate predictions of aerosol radiative effects.^20,43^

The impact of the representation of surfactant partitioning on box and global circulation models has also been investigated. Prisle et al. compared three descriptions of surfactant partitioning in aerosol droplets to the assumption of a pure water surface tension in a global circulation model.^43^ They found that the method of representing surface partitioning and aerosol surface tension in climate models greatly impacts the results. Including surfactant partitioning, the five-year mean shortwave cloud radiative forcing could change from −1.27 to +0.17 W/m^2^ compared to the pure water surface tension assumption.^43^ Lowe et al. compared the assumption of a pure water surface tension to a condensed film description of aerosol surface tension for marine, boreal, and nascent ultrafine mode aerosol compositions in a box model.^44^ Including the changing surface composition increased the cloud droplet number concentration for each case, with the most significant changes predicted to be in the boreal and nascent ultrafine mode aerosol compositions.^44^ The results from both of these investigations highlight the importance of understanding aerosol surface tension and partitioning of surface active components between the droplet surface and bulk.

In this work, we compare surface tension predictions from the Monolayer Model and the Simple Kinetic Model developed by Alvarez et al.^29^ and directly compare these predictions to experimentally measured microscopic droplet surface tensions^42^ for 12 unique surfactant–cosolute systems spanning a wide range of surface-active properties and droplet compositions and sizes. The Simple Kinetic Model determines the effective droplet bulk surfactant concentration from a mass balance calculation where the kinetic parameters are retrieved by applying the Langmuir isotherm to macroscopic solution data and from knowledge of the SA-V ratio of the droplet.^29^ In contrast, the Monolayer Model uses a two-step iteration to determine the partitioning between the surface and bulk for all species in a droplet.^20^ To our knowledge, the Simple Kinetic Model, a model that has a numerical solution and is straightforward to implement, has not been compared to other partitioning models. Additionally, this contribution serves as the first quantitative comparison of the ability of different partitioning models to predict the strength of surfactant partitioning, as well as size-dependent partitioning for ternary droplets containing strong surfactants.

## Methods

Predictions from the Simple Kinetic Model^29^ for 6–9 μm radius droplet surface tensions are directly compared to Monolayer Model^20^ predictions and experimentally measured droplet surface tensions previously reported by Bain et al.^42^ Droplets contain a cosolute, either 0.5 M NaCl or 0.9 M glutaric acid, and a nonionic surfactant [octyl-β-d-1-thioglucopyranodide (OTG), Tween20, or a linear poly(oxyethylene) alkyl ether (CmEm: C16E8, C14E6, C12E5, and C10E8)]. Table S1 contains the chemical structures of these surfactants, as well as their molar masses and their critical micelle concentrations (CMC) in binary aqueous solution.

Both models utilize the same macroscopic surface tension data^42^ as input to predict the partitioning. Direct comparison between the two models is ensured by utilizing the same temperature (298 K) and by setting the minimum droplet surface tension to the minimum surface tension from the macroscopic measurements in both cases.

### Droplet Surface Tension Modeling

#### Monolayer Model

The Monolayer Model uses a semiempirical relationship (eq 1) to iteratively calculate the compositions of the droplet bulk (superscript b)  and surface (superscript *s*)  from the droplet surface tension, σ, parametrized in terms of the composition of the bulk (left side of eq 1) and weighted by the volumes of individual components in the surface (right side of eq 1).  and  are the bulk and surface mole fractions, respectively, corresponding to molar amounts  and , ν_*i*_ are the molecular volumes, and σ_*i*_ are pure compound surface tensions for each droplet component *i*.^20^
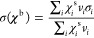
1

Mass conservation for the total number of moles, , is imposed as a constraint for each of the *i* components in the droplet. For the surface phase, the mass balance is
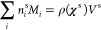
2where *M*_*i*_ is the molar mass (g/mol) of each component *i*, ρ(**χ**^s^) is the bulk density of the liquid monolayer, and the surface monolayer volume is *V*^s^ = 4π[*r*^3^ – (*r* – δ)^3^]/3, where *r* is the droplet radius. The surface monolayer thickness, δ, is estimated as the average diameter of molecules that make up the surface.
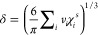
3

The Monolayer Model requires inputs of composition-dependent surface tension and density for all mixtures and pure components in the system of interest. The volume of each molecule at the interface is calculated as the volume of a sphere, constrained by its pure component density.

Macroscopic surface tension data were parametrized with a modified Szyszkowski–Langmuir equation for the left side of eq 1 using eq 4a for systems containing surfactant OTG and eq 4b for systems containing all other surfactants. The modified Szyszkowski–Langmuir equations are semiempirical equations, which have been found to capture the shape of changing surface tension with surfactant concentration. This fit implicitly includes any intermolecular interactions between the cosolute and surfactant. Equations 4a and 4b (which differ only by scaling certain terms) are necessary due to the large differences in surfactant concentrations required to reach the CMC for the different systems (spanning from μM for C16E8 to tens of mM for OTG). Concentration units of molar for both the surfactant (*C*_surf_) and cosolute (*C*_co_) should be used in eqs 4a and 4b. Fitting parameters are listed in Table 1. (Note that in eqs 4a and 4b, *b*_2_ is multiplied by the surfactant concentration, correcting a typographical error in eq 11 in the Supporting Information of ref (42).) 
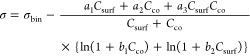
4a
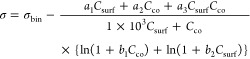
4b

**Table 1:** Fitting Parameters and Standard Error of Regression (SER) for the Szyszkowski–Langmuir Parameterization of Macroscopic Surface Tension Data Using Eqs 4a and 4b Used as Input for the Monolayer Model^a^^42^

Surfactant	*a*_1_ (Nm^-1^)	*a*_2_ (Nm^-1^)	*a*_3_ (NM^-1^m^-1^)	*b*_1_ (M^–1^)	*b*_2_ (M^–1^)	SER (Nm^-1^)
0.9 M Glutaric Acid						
C16E8	87.343	0.0061122	–100.95	–0.2715400	3470100	0.000798
C14E6	108.71	0.0045747	–101.43	0.0060938	9231100	0.000474
C12E5	103.64	0.0054407	–107.07	0.0116870	659320.0	0.000413
C10E8	102.04	0.0034107	–108.84	0.0035685	313990.0	0.000691
Tween20	50.254	0.0064715	–55.274	–0.0798380	748830.0	0.001310
OTG	0.033005	0.0114380	–0.07444	0.0376540	773.4800	0.000468
0.5 M NaCl						
C16E8	251.00	0.0072440	–482.30	–0.044224	17006000.0	0.00228
C14E6	250.00	0.0058200	–499.00	–0.037000	20856000.0	0.00249
C12E5	251.14	0.0085308	–497.73	–0.063328	3771500.00	0.00138
C10E8	27.461	0.0080942	–45.080	0.073147	332180.000	0.00050
Tween20	1239.3	0.0162900	–2521.4	0.123440	331960.000	0.00190
OTG	1.2944	0.0107920	–2.4113	–0.099817	6872.10000	0.00110

a Note that typographical errors in the units from ref (42) have been corrected.

In eqs 4a and 4b, σ_bin_ is the surface tension of the binary cosolute–water solution obtained from the literature (Gaman et al.^45^ for water–glutaric acid and Vanhanen et al.^46^ for water–NaCl) and *a*_1_, *a*_2_, *a*_3_, *b*_1_, and *b*_2_ are the fitting parameters. The surface tensions of pure, nonaqueous surfactants are approximated by the surface tensions at the CMC of the surfactant in macroscopic aqueous solution, σ_CMC_. This approximation assumes that a pure monolayer with  has formed at the CMC. Surface tensions at the CMC used here can be found in Table S2. The surface tension and density of the pure component cosolutes are extrapolated from the correlations presented by Janz (NaCl)^47^ and Gaman et al. (glutaric acid).^45^ Liquid phase densities ρ(**χ**^s^) for ternary water–surfactant–cosolute solutions are estimated by combining ideal pseudobinary mixtures of aqueous glutaric acid/NaCl and surfactant, using the method of Kodama and Miura.^48^ Densities for mixtures of aqueous glutaric acid or NaCl are obtained from available literature.^49,50^ The density of the surfactant is taken from a measurement of a concentrated solution that is not available from the supplier. A detailed description of the model framework can be found in the original work of Malila and Prisle.^20^

#### Simple Kinetic Model

The Simple Kinetic Model developed by Alvarez et al. uses a mass balance for surfactant partitioned to the interface and dissolved in the bulk in combination with an isotherm model to express the depleted bulk concentration at equilibrium (eq 5).^29^
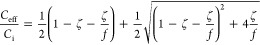
5

The depleted bulk concentration (*C*_eff_) is normalized by the initial bulk concentration (*C*_i_, i.e., total surfactant concentration) and is written as a function of two dimensionless parameters, *f* and ζ. For the case of a spherical droplet with surfactant dissolved in the interior^29^
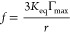
6
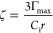
7where *r* is the radius of the droplet, Γ_max_ is the maximum surface excess, and *K*_eq_ = β/α is the equilibrium partitioning constant (i.e., the ratio of adsorption (β) and desorption (α) rate constants), which has units of m^3^/mol.^51^

Macroscopic equilibrium surface tension data sets^42^ provide surface tension data at concentrations below and above the CMC for each surfactant system. Each data set was fit in the region of decreasing surface tension with increasing surfactant concentration to the Langmuir isotherm and equation of state (eqs 8 and 9) to determine Γ_max_ and *K*_eq_.^51^ Fit parameters are listed in Table 2.
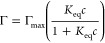
8
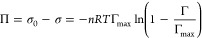
9

**Table 2:** Macroscopic Surface Tension Data Isotherm Fit Parameters^42^ Used as Input for the Simple Kinetic Model (Langmuir Isotherms, Eqs 8 and 9)^a^

	0.9 M Glutaric Acid			0.5 M NaCl		
Surfactant	Γ_max_ × 10^6^ (mol/m^2^)	*K*_eq_ (m^3^/mol)	Molecular Footprint (Å^2^)	Γ_max_ × 10^6^ (mol/m^2^)	*K*_eq_ (m^3^/mol)	Molecular Footprint (Å^2^)
C16E8	5.4 ± 0.6	491 ± 99	31 ± 4	8 ± 1	1063 ± 247	20 ± 3
C14E6	2.09 ± 0.07	5751 ± 724	80 ± 3	2.9 ± 0.2	36,784 ± 12426	58 ± 4
C12E5	2.41 ± 0.05	469 ± 35	69 ± 2	3.5 ± 0.2	3466 ± 592	47 ± 2
C10E8	1.73 ± 0.08	133 ± 25	96 ± 4	2.2 ± 0.1	1254 ± 325	74 ± 4
Tween20	2.7 ± 0.2	537 ± 108	62 ± 4	4.7 ± 0.4	563 ± 115	36 ± 3
OTG	4.0 ± 0.1	0.96 ± 0.06	42 ± 1	4.8 ± 0.2	5.9 ± 0.7	34 ± 1

a Uncertainties in Γ_max_ and *K*_eq_ are standard errors determined during least squares fitting. Uncertainty on the molecular footprint is calculated from the percent uncertainty on Γ_max_.

In the Langmuir isotherm, Γ is the equilibrium surface excess at a specific surfactant concentration, Γ_max_ is the maximum surface excess, *K*_eq_ is the ratio of surface adsorption to desorption rate constants (with units m^3^/mol), *c* is the surfactant concentration, Π is the surface pressure and is defined as the difference between the solvent surface tension (σ_0_, here the surface tension when the surfactant concentration is equal to zero) and the equilibrium surface tension at a given surface excess (σ), *R* is the gas constant, *T* is the temperature (set to 298 K), and *n* is the van’t Hoff factor for the surfactant at the surface. For nonionic surfactants, *n* = 1. The Langmuir isotherm assumes every surfactant adsorption site is equivalent, the probability for adsorption at an empty site is independent of the occupancy of neighboring sites, and there are no intermolecular forces or interactions between monomers adsorbed at the interface.^51^ The Langmuir isotherm also does not include interaction parameters to account for nonideal interactions between the surfactant and cosolute.

In this work, the surface tension when no surfactant is present is set to 62.5 mN/m for ternary aqueous mixtures with 0.9 M glutaric acid and 73.0 mN/m for ternary aqueous mixtures with 0.5 M NaCl. These values are the expected surface tensions for these solutions without the presence of any surfactant.^45,47^ The model results are not sensitive to small differences (∼1 mN/m) in σ_0_. To predict the surface tension in a droplet with depleted surfactant concentration due to bulk-surface partitioning, *c* in eq 8 is replaced with *C*_eff_, and eqs 8 and 9 are solved for surface tension. Note, as droplet radius increases, *C*_eff_ approaches *C*_i_ and the predicted surface tension approaches the macroscopic surface tension from the Langmuir isotherm. When the predicted surface tension becomes equal to the average surface tension measured for macroscopic solutions at concentrations greater than the CMC (Table S2), the surface tension is set to be equal to this average value for all larger surfactant concentrations. The minimum surface tension for a droplet in the micron size range is not expected to differ from the minimum surface tension of a macroscopic solution.^52^

### Model-Measurement and Model–Model Comparisons

The agreement between each model’s predictions and the experimentally determined surface tensions is quantified with the mean absolute error (MAE) and is defined as
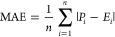
10where *P_i_* and *E*_*i*_ are the model prediction and experimental surface tension, respectively, for data point *i*, and *n* is the total number of data points. Because the model predictions have two boundaries (6 and 9 μm radius droplets), one would expect all droplet measurements within this size range to fall within these boundaries if the model and experiment were in complete agreement. The residual, *P*_*i*_ – *E*_*i*_, is taken to be zero if , where  and  are the model predictions for 6 and 9 μm radii, respectively. If , the residual is ; if , the residual is . Because droplet coalescence can induce tighter packing of surfactants at concentrations beyond the apparent CMC, leading to a lower surface tension than equilibrium, the MAEs were calculated after removing data points, where the concentration is greater than the average of the effective droplet CMCs, predicted by the model for 6 and 9 μm radii.

To compare the models against each other, the mean absolute scaled error (MASE) is calculated as the ratio of the MAE between the experimental data and each model’s predictions.
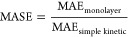
11

When MASE = 1, the two models are equally good or poor at predicting the experimental data. If MASE = 0.5, the Monolayer Model is twice as good as the Simple Kinetic Model, whereas if MASE = 2, the Simple Kinetic Model is twice as good at predicting the experimental data.

## Results and Discussion

### Surface Tensions of 6–9 μm Radius Droplets

Surface tension predictions for 6–9 μm radius droplets using the Monolayer and Simple Kinetic Models were compared to experimentally measured surface tensions of 6–9 μm radius aerosol droplets previously reported.^42^ These surfactants span a wide range in surface active properties and have CMCs between 0.0033 and 20.8 mM. Here, we relate the CMC with surfactant strength, with stronger surfactants having lower CMCs. The range of surface activity investigated is consistent with those of environmental surfactants and extracts of aerosol samples.^2,4,53^ Simple Kinetic Model predictions were calculated as described in the Methods section.

**Figure 1:**
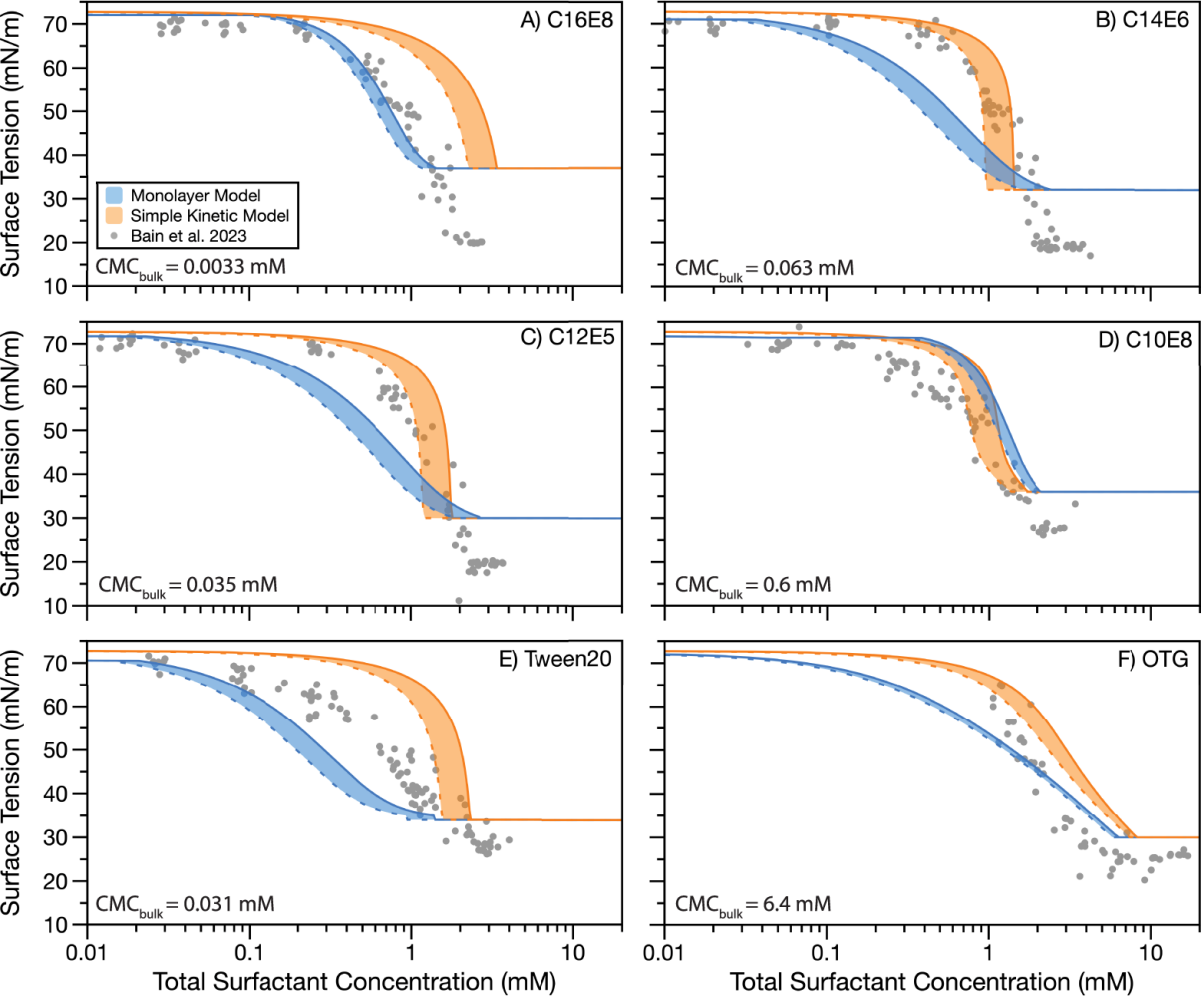
Comparison of surface tension predictions from the Monolayer Model (blue) and Simple Kinetic Model (orange) to droplet surface tensions for droplets of radius 6–9 μm.^42^ Solid model boundary shows 6 μm prediction, and dashed model boundary shows 9 μm prediction. Droplet measurements and model predictions are for ternary aqueous aerosols containing 0.5 M NaCl and (A) C16E8, (B) C14E6, (C) C12E5, (D) C10E8, (E) Tween20, and (F) OTG. CMC_bulk_ in each panel indicates the concentration at which the surface tension of a macroscopic solution is no longer reduced with the addition of more surfactant.

**Figure 2:**
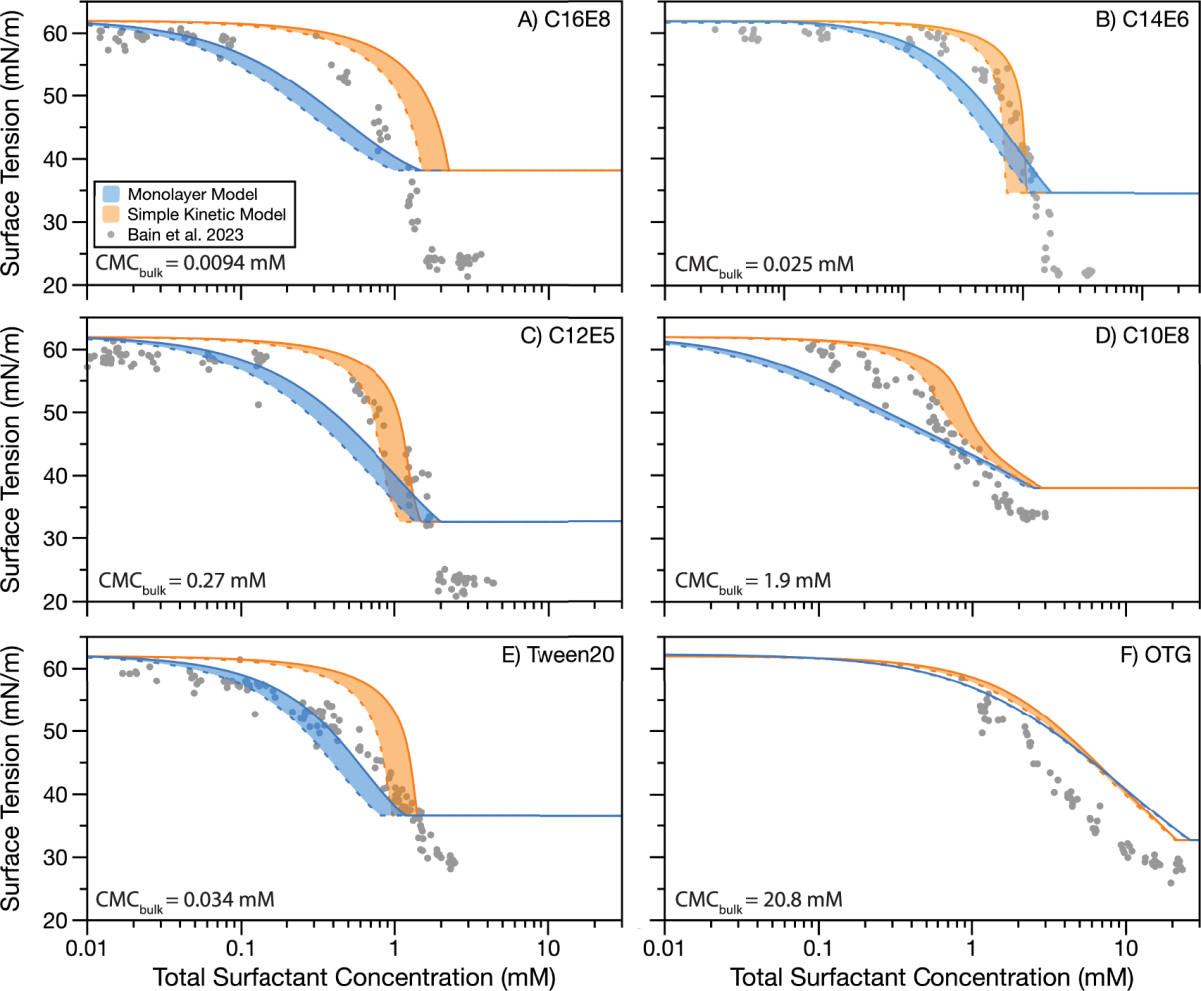
Comparison of surface tension predictions from the Monolayer Model (blue) and Simple Kinetic Model (orange) to droplet surface tensions for droplets of radius 6–9 μm.^42^ Solid model boundary shows 6 μm prediction, and dashed model boundary shows 9 μm prediction. Droplet measurements and model predictions are for ternary aqueous aerosols containing 0.9 M glutaric acid and (A) C16E8, (B) C14E6, (C) C12E5, (D) C10E8, (E) Tween20, and (F) OTG. CMC_bulk_ in each panel indicates the concentration at which the surface tension of a macroscopic solution is no longer reduced with the addition of more surfactant.

Figure 1 plots the predicted droplet surface tension as a function of total surfactant concentration (which accounts for the moles of surfactant at the droplet surface and in the droplet bulk) for each of the six surfactants with NaCl as the cosolute, whereas Figure 2 plots predicted and measured droplet surface tensions for the same surfactants with glutaric acid as the cosolute. Figures 1 and 2 qualitatively demonstrate that the predicted surface tensions from the Monolayer and Simple Kinetic Models are broadly similar to each other and are close to the experimentally measured values. Nonetheless, there are some differences between the two sets of model predictions. The Monolayer Model almost always predicts a more gradual change in surface tension with increasing total surfactant concentration compared to the Simple Kinetic Model, resulting in a lower surface tension than the Simple Kinetic Model at the same total surfactant concentration. In contrast, both models overlap the experimental data for the two systems: C10E8 in 0.5 M NaCl (Figure 1D) and OTG in 0.9 M glutaric acid (Figure 2F). The system reported in Figure 2F does not exhibit bulk depletion in the experimentally investigated droplet size range, so both models are expected to provide similar outputs, as they should both tend to the underlying macroscopic data.

In Figures 1 and 2, neither model more consistently matches the experimental data than the other. For some systems, the Monolayer Model overlaps better with the experimental data (e.g., Figures 1A and 2E), while for other systems, the Simple Kinetic Model more closely predicts the experimental data (e.g., Figures 1B and 2B). There are also systems where the experimental data fall between the two sets of model predictions (e.g., Figures 1E and 2A). In the case of OTG in 0.9 M glutaric acid OTG (Figure 2F), both models overpredict the measured surface tensions. This observation is due to the measured droplet surface tension being slightly lower than that of the macroscopic data used to parametrize the models due to the formation of a condensed film on the surface of the composite droplet upon coalescence.^41,42^ When the total surfactant concentration becomes large enough for the CMC to be reached in the droplet bulk, both sets of model predictions are higher than those of the experimental measurements. The minimum surface tension in both models is limited by the minimum surface tension of the macroscopic systems. Droplet measurements using the coalescence method have been found to underpredict the surface tension in this region due to the presence of a condensed film formed upon droplet coalescence.^41,42^

### Constant Droplet Radius and Changing Surfactant Concentration

Here, a more quantitative comparison of the Monolayer and Simple Kinetic Model predictions to the experimental data is provided. Specific comparisons include the total amount of surfactant required to reach the CMC in the droplet bulk, the size-dependence of surface tension predictions, and absolute comparisons of measurement-model agreement.

**Figure 3:**
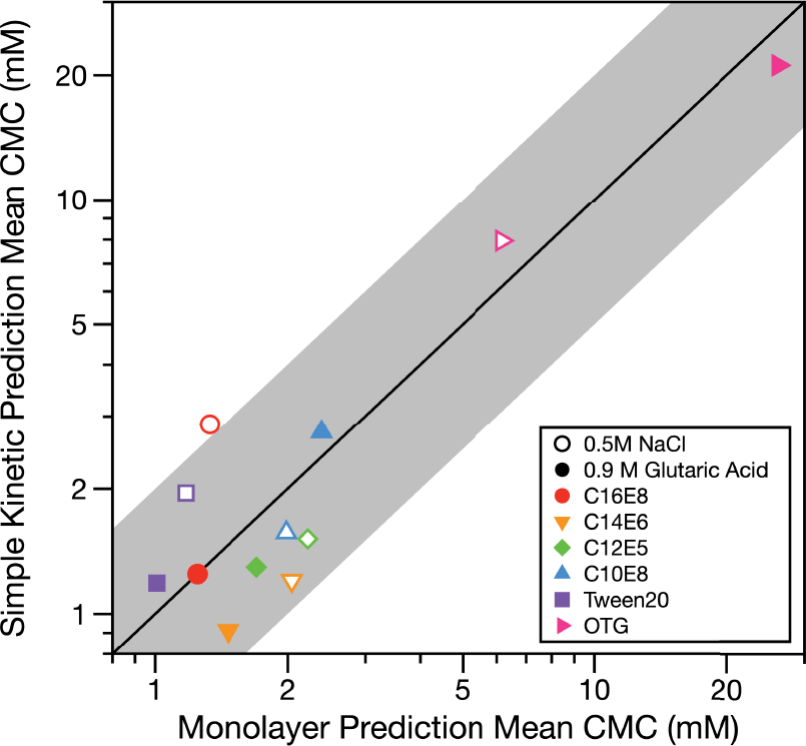
Comparison of the mean apparent CMC predicted for 6–9 μm radius droplets containing 0.5 M NaCl (open markers) or 0.9 M glutaric acid (closed markers). The black diagonal line shows 1:1 where the Simple Kinetic and Monolayer Models would predict the same apparent CMC, and the gray shaded region shows a factor of 2 difference. Apparent CMCs from the model predictions have been plotted on a logarithmic scale for clarity.

The Monolayer and Simple Kinetic Models both predict that similar total surfactant concentrations are required to reach the CMC in the droplet bulk. Figure 3 and Table S3 compare the apparent droplet CMCs across both models. For ease of comparison, the average predicted apparent CMC for droplets between 6 and 9 μm radius has been plotted and tabulated. Only OTG, the least effective surfactant investigated and not expected to exhibit bulk depletion in this size range, has predicted apparent droplet CMCs >3 mM. This observation is consistent with our previous findings that strong surfactants of similar molecular footprint require similar total amounts of surfactant to reduce the surface tension.^42^ No obvious trends are observed (e.g., one model predicting higher apparent CMCs than the other or trends with cosolute or surfactant strength). The difference between the two models is always within a factor of ∼2. The models generally have closer agreement for glutaric acid cosolute than NaCl cosolute. This result is consistent with the salting out of surfactant in NaCl solution being handled better by the Monolayer Model, which implicitly accounts for nonideality, compared to the pseudoideal approach of the Simple Kinetic Model. For example, the Monolayer Model predicts apparent CMCs closer to the experimentally observed total surfactant concentration required to reach the minimum surface tension (Table S3) than the Simple Kinetic Model. Nonetheless, both models predict apparent droplet CMCs within a factor of ∼2 of the droplet measurement estimates.

The strength of size-dependent depletion in the 6 and 9 μm radius range predicted by the two partitioning models is investigated by integrating the area between the two model prediction boundaries (Figure S1 and Table S4). The integrated prediction areas of the two models for the four strongest surfactants (i.e., the surfactants with the lowest CMCs in macroscopic solution: C16E8, C14E6, C12E5, and Tween20) with a glutaric acid cosolute are nearly identical. For OTG and C10E8 (two weaker surfactants), the Simple Kinetic Model has a larger integrated area than the Monolayer Model, resulting from a steeper predicted gradient in bulk depletion with droplet size. Although the six surfactants studied span a wide range in surface activity (orders of magnitude in macroscopic CMC), the extent of depletion within the size range investigated here does not scale with the surfactant strength. There is a general trend of surfactants in 0.5 M NaCl having a larger integrated area than the same surfactant in 0.9 M glutaric acid. The spread in the experimentally determined droplet surface tensions is generally in agreement with the models; however, this spread is in part due to measurement uncertainty. Using the Simple Kinetic Model, the depletion strength can also be shown by using the normalized effective bulk concentration as a function of 1/ζ, where ζ is the maximum fractional potential mass lost to the interface. Figure S2 shows this depletion for each surfactant in droplets of 6 and 9 μm radii, spanning the total surfactant concentration range in the droplets. There is a clear difference between depletion for droplets containing OTG and droplets containing other surfactants in the concentration and size space explored here.

Next, the agreement between each model and the reference droplet data is investigated. The MAEs between the data^42^ and the two models were calculated using eq 10. MAEs are shown in Table S5 and Figure S3. MAEs span 3–8 mN/m for the Monolayer Model and 2–15 mN/m for the Simple Kinetic Model. The two models have MAEs within a factor of ∼2 for surfactants in glutaric acid. When the cosolute is NaCl, the MAEs exhibit a larger amount of scatter.

**Figure 4:**
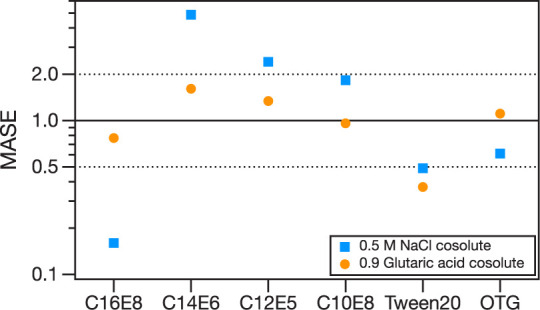
Mean absolute scaled error (MASE) comparing the goodness of fit between the Simple Kinetic and Monolayer Models to the experimental data.^42^ An MASE of 1 (solid black line) indicates that both models describe the experimental data equally well. A MASE of 2 indicates that the Simple Kinetic Model agrees with the experimental data twice as well as the Monolayer Model, and an MASE of 0.5 indicates that the Monolayer Model agrees with the experimental data twice as well as the Simple Kinetic Model. MASE values of 2 and 0.5 are indicated with dotted lines.

MASE is used to compare the goodness of fit of the two models to the droplet data (Figure 4 and Table S5). Overall, the two models agree with the droplet data as well or as poorly as with one another, with MASE falling between 0.5 and 2. There are only a few cases where one model agrees with a data set more than twice as well as the other. For C16E8 in NaCl, the Monolayer Model agrees with the data set substantially better than the Simple Kinetic Model (MASE = 0.16), whereas for C14E6 in NaCl, the Simple Kinetic Model significantly outperforms the Monolayer Model (MASE = 4.88). We note that for C16E8 in NaCl, Γ_max_ retrieved from the Langmuir isotherm fit of the macroscopic data (Table 2) had the largest uncertainty of all investigated systems and that the Szyszkowski–Langmuir parametrization for C14E6 in NaCl had the largest standard error of regression (Table 1) of all systems. In Figure 4, the absolute value for MASE for a given surfactant with NaCl cosolute is larger than that with glutaric acid cosolute except Tween20. The isotherm and parametrization of macroscopic surface tension data for surfactants with NaCl cosolute have larger fitting errors than those for surfactants with glutaric acid cosolute. Therefore, the quantitative ability of these models to predict aerosol droplet surface tension depends considerably on the quality of the macroscopic surface tension data and the isotherm. This observation motivates careful measurements of macroscopic solution surface tensions for atmospherically relevant surfactant systems.

### Changing Droplet Radius with Constant Surfactant Concentration

**Figure 5:**
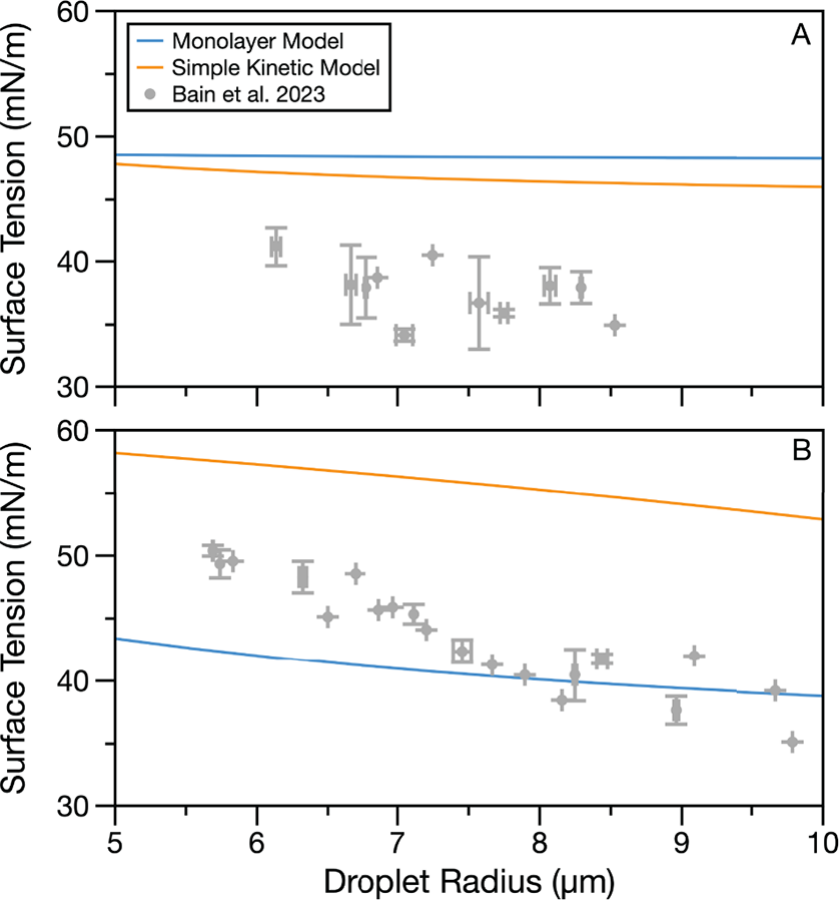
Surface tension predictions from the Monolayer and Simple Kinetic Models for (A) 4 mM OTG and 0.9 M glutaric acid and (B) 0.8 mM C16E8 and 0.9 M glutaric acid as a function of droplet radius. Predictions are compared to reference data.^42^

Size-dependent surface tension predictions from the Monolayer and Simple Kinetic Models for droplets having a fixed composition are now compared to the reference data sets for 4 mM OTG and 0.8 mM C16E8, each with 0.9 M glutaric acid colsolute.^42^Figure 5 shows this comparison. Qualitatively, the two models agree with one another and predict a similar change in surface tension with the droplet radius over the 5–10 μm size range. In Figure 5A (4 mM OTG in glutaric acid), the two model lines predict higher surface tensions than the measurements and only a very small change in surface tension with the droplet size (0.26 mN/m over the 5–10 μm radius size range for the Monolayer Model and 1.8 mN/m for the Simple Kinetic Model), which qualitatively agrees with the trend in the droplet data. In Figure 5B (0.8 mM C16E8 in glutaric acid), the Monolayer Model passes through the measurements, but neither model predicts the nearly 15 mN/m change in surface tension observed across 5–10 μm radius droplets. The models again qualitatively agree with one another, with the Monolayer Model predicting a change of 4.6 mN/m over the 5–10 μm radius size and the Simple Kinetic Model predicting a change of 5.3 mN/m. The discrepancy between the model predictions and the experimental data may be in part due to systematic undermeasurement associated with the droplet coalescence approach in this concentration range, reducing the measured droplet surface tension relative to its equilibrium value.^41,42^ The slightly larger change in surface tension with size predicted by the Simple Kinetic Model in Figure 5 for both C16E8 and OTG is consistent with that model’s larger integrated area between the 6 and 9 μm radius (Figure S1 and Table S4).

### Model Predictions for Submicron Droplets

**Figure 6:**
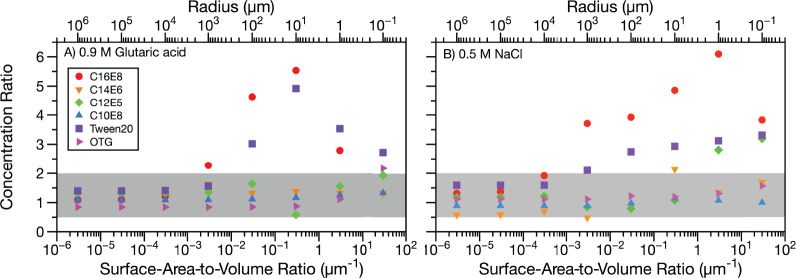
Concentration ratio (the total concentration of surfactant required to reach the CMC in the droplet bulk predicted by the Monolayer Model divided by the total concentration of surfactant required to reach the CMC in the droplet bulk predicted by the Simple Kinetic Model) as a function of droplet surface-area-to-volume ratio (and radius) for C16E8, C14E6, C12E5, C10E8, Tween20, and OTG in solution with (A) 0.9 M glutaric acid and (B) 0.5 M NaCl cosolute. The gray shaded region shows a factor of 2 difference between the two models.

The measurements for 5–10 μm radius droplets establish that both models perform with largely similar degrees of accuracy when benchmarked against experiments. However, atmospheric CCN are typically smaller than this size range.^54^ The predictions from the two models for the total surfactant concentration required to reach the minimum surface tension in the droplet bulk (i.e., reach a full monolayer surfactant surface coverage) as a function of SA-V ratio are now compared to determine differences in these predictions for submicron aerosols. Figure 6 shows the concentration ratio (defined in eq 12) for droplets with radii spanning 10^6^ μm (macroscopic systems) to 10^–1^ μm (ambient aerosols).
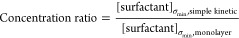
12

In Figure 6, when the SA-V ratio is small (i.e., the droplet radius is large), the two models predict similar total surfactant concentrations to reach the CMC in the droplet bulk (giving a concentration ratio close to one for both cosolutes). This observation is expected since both models converge to macroscopic solution values at large droplet sizes. As the SA-V ratio increases and depletion starts to come into effect (∼1000 μm radius), there is generally still good agreement between the two models, and the concentration ratio is between 0.5 and 2 for most systems, even at the largest SA-V ratios. There are, however, some exceptions to this trend, particularly at smaller droplet radii. In Figure 6A, in mixtures with glutaric acid, the Simple Kinetic Model predicts 3–6 times more surfactant required to reach the minimum surface tension in the droplet bulk than the Monolayer Model for C16E8 and Tween20 surfactants in the 1–10^3^ μm radius range. Interestingly, there is a maximum in the divergence between the two models at a 10 μm radius, which largely disappears as the radius is reduced to 0.1 μm. In Figure 6B (NaCl cosolute), as the droplet radius decreases, the Simple Kinetic Model begins to predict more surfactant is required to reach the minimum surface tension than the Monolayer Model for C16E8, Tween20, and, at the smallest radii, C12E5. In the case of C16E8 with NaCl, the same trend is observed as with glutaric acid, where the divergence between the two models reaches a maximum and then decreases, whereas for Tween20 and C12E5, a more gradual change in the concentration ratio with decreasing droplet size is observed. The increased occurrence of discrepancies between the two models for NaCl cosolute is consistent with the larger differences between the models’ integrated areas and MAEs for surfactants in NaCl cosolute compared to glutaric acid cosolute.

The large concentration ratios, resulting from the Simple Kinetic Model predicting higher total surfactant concentrations to reach the minimum surface tension than the Monolayer Model for some systems, arise from differences in the bulk depletion that these two models predict. Droplets with radii larger than ∼100 μm exhibit little bulk depletion, and both models return the underlying macroscopic isotherm. At a radius of 0.1 μm, both models predict a strong depletion. The poorest agreement between the two models is often found for intermediately sized droplets. These differences are a result of the two models predicting different droplet sizes for the onset of depletion, resulting from a difference in their predictions of the surfactant partitioning strength. Nevertheless, for 77% of the simulations in Figure 6, the Monolayer and Simple Kinetic Models predict the concentration required to reach the minimum surface tension in a droplet to within a factor of 2 of one another. We note that C16E8 is the most surface active surfactant considered here and due to its very low CMC (<10 μM in 0.9 M glutaric acid and 0.5 M NaCl solutions^42^), it has the largest uncertainty associated with the macroscopic isotherm used to fit the partitioning models. Furthermore, Tween20 has a branched structure, and its partitioning may not be well represented by the assumptions made in the Langmuir adsorption isotherm, which underpins the Simple Kinetic Model. The differences in total concentration required to reach the droplet bulk CMC for C16E8 and Tween20 systems using the Monolayer and Simple Kinetic Models again show that surface tension predictions are sensitive to the partitioning model, highlighting the importance of selecting a partitioning model that has been tested against experimental data. Partitioning models should be validated by droplet surface tension data in the size range where bulk concentration depletion begins to occur before being implemented in larger scale cloud or climate models. The intermediate size range (1–100 μm) is accessible by most single droplet surface tension measurement techniques^35,36,38−41,55^ and extends to the largest ambient aerosol droplet sizes.

### Maximum Surface Concentrations

**Figure 7:**
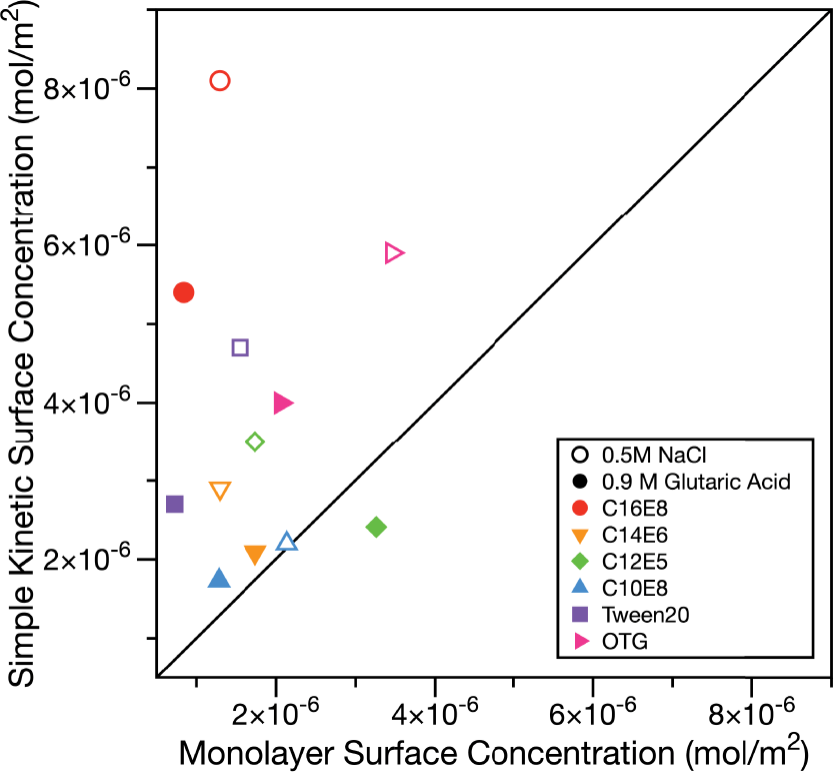
Comparison of the surfactant surface concentration when the minimum surface tension is reached. Black diagonal line shows 1:1 where the Simple Kinetic and Monolayer Models predict the same maximum surface concentration. Error bars have been omitted for the sake of clarity. Uncertainties are provided in Table 3.

Finally, the differences between the maximum surface concentration predicted by the Simple Kinetic and Monolayer Models are investigated. Understanding surface concentrations is important when considering the reactivity at droplet interfaces. In the Simple Kinetic Model, the maximum surface concentration is retrieved directly from the fit of macroscopic data to the Langmuir isotherm. Γ_max_ values for each system are provided in Table 2. The maximum surface concentration prediction from the Monolayer Model is calculated using the mole fraction of the surfactant at the droplet interface (a model output), the total surfactant concentration, and the surface area of the droplet (see Text S1 for further description). The Monolayer Model predicts the surface layer to be only composed of the surfactant, and so this surface concentration can be thought of as a packing of the molecules at the interface. This calculation is performed at every droplet radius in Figure 6 to determine an average and standard deviation. The resulting surface concentrations are tabulated in Table 3, and Figure 7 compares the predicted maximum surface concentrations of the two models.

**Table 3:** Maximum Surface Concentration and Corresponding Molecular Footprint at the Surface from Monolayer Model Predictions^a^

	0.9 M Glutaric Acid		0.5 M NaCl	
Surfactant	Surface Concentration (×10^6^ mol/m^2^)	Molecular Footprint (Å^2^)	Surface Concentration (×10^6^ mol/m^2^)	Molecular Footprint (Å^2^)
C16E8	0.8 ± 0.4	222 ± 67	1.3 ± 0.2	131 ± 20
C14E6	1.7 ± 0.8	97 ± 11	1.3 ± 0.8	195 ± 136
C12E5	3 ± 1	64 ± 39	3 ± 1	78 ± 42
C10E8	1.29 ± 0.06	129 ± 1	2.14 ± 0.07	78 ± 3
Tween20	0.7 ± 0.1	235 ± 47	1.6 ± 0.2	109 ± 11
OTG	2.1 ± 0.3	82 ± 15	3.5 ± 0.1	48 ± 2

a Presented data are an average and standard deviation from 0.1 to 1 × 10^6^ μm radius droplets simulated in Figure 6

The Monolayer Model predicts lower maximum surface concentrations than the Simple Kinetic Model for every system except C12E5 in 0.5 M NaCl. The maximum surface concentrations can be converted to molecular footprints at the interface (footprint = *N*_A_/Γ_max_, Tables 2 and 3 and Figure S4). The smaller maximum surface concentrations predicted by the Monolayer Model correspond to larger molecular footprints at the interface. These differences in maximum surface concentration and molecular footprint can be understood based on each modeling approach. The Simple Kinetic Model uses the Langmuir isotherm to determine the maximum surface concentration. Surface concentrations determined from the Langmuir isotherm fit in Table 2 show no trend with molecular weight. For example, the largest molecular weight surfactant, Tween20 (1227.53 g/mol), and the lowest molecular weight surfactant, OTG (308.43 g/mol), have intermediate Γ_max_ values in each cosolute. The Monolayer Model uses an effective spherical volume to describe each surfactant. This approach results in a larger variation in total surface concentration and molecular footprint at the interface than predicted by the Langmuir isotherm. The Monolayer Model also predicts larger differences between the maximum surface coverage (and molecular footprint) for a single surfactant in solution with different cosolutes than the Simple Kinetic Model because nonideal interactions are implicitly included in the model and cosolute partitioning is treated simultaneously. The increased chemical information included in the Monolayer Model compared to the Simple Kinetic Model leads to larger differences in the maximum surface concentration and molecular footprint; however, both models are based on a simplified concept of the surface.

## Conclusion

Comparisons of the Monolayer Model and Simple Kinetic Model surface tension predictions for surfactant-containing aerosol droplets enable assessment of their relative strengths and weaknesses as well as the underlying factors contributing to their differences. The surfactant systems investigated here serve as proxies for nonionic surfactants found in aerosols with CMCs spanning the full range detected for surfactants in the atmosphere. Furthermore, we have investigated surfactants in mixtures with NaCl (a laboratory proxy for sea salt and a component of ambient aerosols) and glutaric acid (a laboratory proxy for highly oxidized organic matter found in aerosols).

Partitioning models have previously been compared to measurements of critical supersaturation, which do not provide sufficient information to disentangle the extent of surface tension reduction and surface tension size-dependence that a model predicts. Our comparisons demonstrate that the Monolayer and Simple Kinetic Models perform similarly (often within a factor of 2) with respect to predictions of onset of depletion as well as agreement with size- and composition-dependent experimental data. The models exhibit more substantial differences in some cases, mainly for the strongest surfactants and for droplets containing NaCl. Model disagreements were most significant for systems where isotherm fits or parametrization errors were largest, highlighting the importance of high quality macroscopic surface tension data sets.

Future work should compare additional partitioning models to aerosol surface tension measurements and investigate a wider range of surfactant types (e.g., ionic, less surface active, biological, and humic-like substances). Validating model predictions against each other and against experimental measurements is essential in order to make confident predictions of droplet physicochemical properties and surface chemistry. Moreover, such validation is needed before incorporation of these surface tension models into cloud parcel models to assess potential aerosol surface tension effects on cloud formation and radiative forcing.

## Data Availability

All data underlying the figures are available in the tables and Supporting Information.

## Supporting Information Available

Graphical comparisons of integrated area between model predictions for 6–9 μm radius droplets, mean absolute errors (MAEs) for model predictions and experimental data, molecular footprints for surfactants, and the Simple Kinetic Model normalized bulk concentration as a function of maximum potential mass loss to the interface; tabulated data for surfactant properties, droplet critical micelle concentrations (CMCs), integrated areas between 6 and 9 μm radius droplet predictions, mean absolute errors (MAEs), and mean absolute scaled errors (MASEs); text describing process to calculate the surface concentration from the Monolayer Model output (PDF)
