# Supplementary material for: Model-Measurement Comparisons for Surfactant-Containing Aerosol Droplets
Source: ACS Earth Space Chem. 2024 Oct 22;8(11):2244–55. doi: 10.1021/acsearthspacechem.4c00199 (PMC11587080; doi:10.1021/acsearthspacechem.4c00199)
Supplement: Supplementary file 1 — sp4c00199_si_001.pdf [file sp4c00199_si_001.pdf]

Supplementary Information for:

Model-Measurement Comparisons for Surfactant-Containing Aerosol Droplets

Submitted to ACS Earth and Space Chemistry

Alison Bain[1,2] Nønne L. Prisle[3] & Bryan R. Bzdek[1,*]

[1]School of Chemistry, University of Bristol, BS8 1TS, Cantock's Close, Bristol, United Kingdom

[2]Department of Chemistry, Oregon State University, 97331, 2100 SW Monroe Ave, Corvallis, Oregon, USA

[3]Center for Atmospheric Research, University of Oulu; Oulu, 90014, Finland

*Corresponding author: b.bzdek@bristol.ac.uk

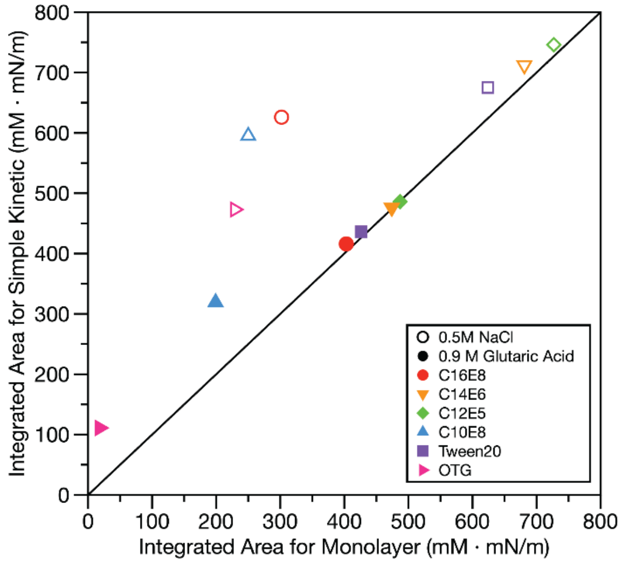

Figure S1: Comparison of the integrated area between the 6 – 9 μm radius droplet model predictions. Black diagonal line shows 1:1 where Simple Kinetic and Monolayer Models would predict the same area between the 6 – 9 μm lines.

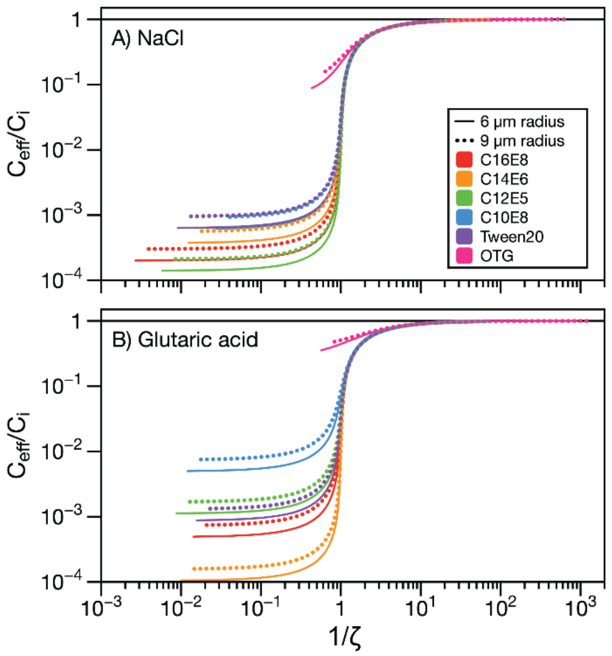

Figure S2: Normalized effective bulk surfactant concentration for surfactant-containing droplets investigated in this study. A) Surfactants with 0.5 M NaCl cosolute. B) Surfactants with 0.9 M glutaric acid cosolute. Solid black lines indicate where the effective bulk concentration is equal to the total concentration and, therefore, no bulk concentration depletion is predicted with the Simple Kinetic Model.

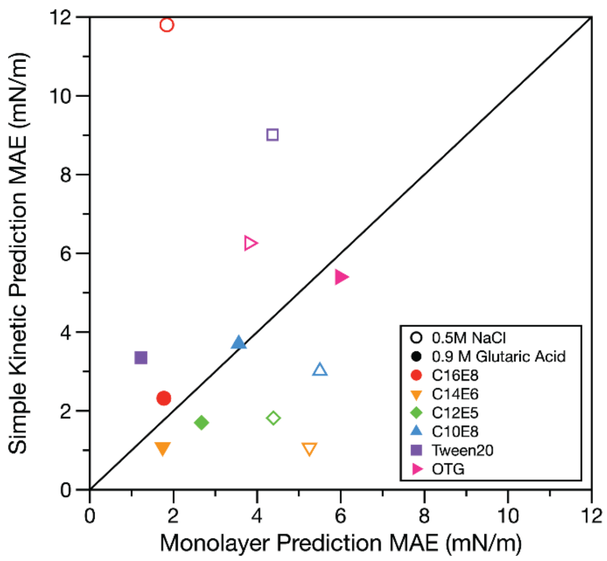

Figure S3: Mean absolute error (MAE) between model predictions and experimental data using only datapoints where the total surfactant concentration is less than the apparent CMC predicted by the model. Black diagonal line shows 1:1 where Simple Kinetic and Monolayer Models would agree with the reference data as well as one another.

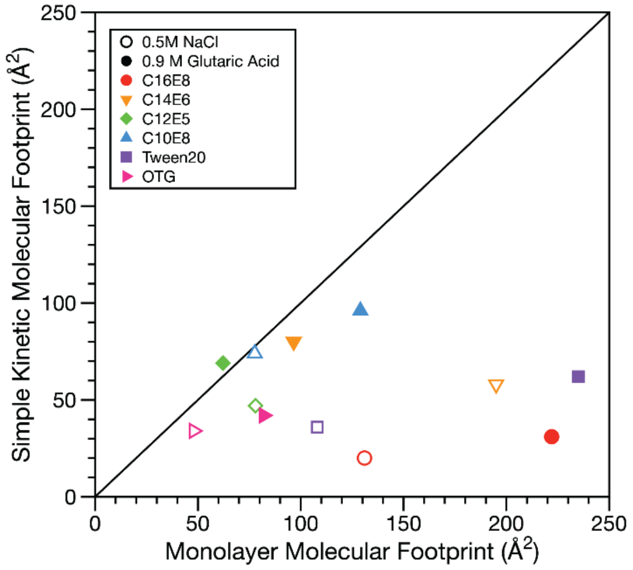

Figure S4: Comparison of surfactant molecular footprint (area) at the interface for the Simple Kinetic and Monolayer Models. Black diagonal line shows 1:1 where Simple Kinetic and Monolayer Models would predict the same molecular footprint.

Table S1: Physical parameters of surfactants investigated in this study.

Surfactant	Structure	Molar mass (g/mol)	CMC in binary aqueous solution (mM)	Density (g/cm^3)
C16E8	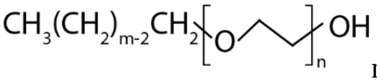 m = 16 n = 8	594.861	0.001-0.016 [1–4]	0.9987 [4]
C14E6	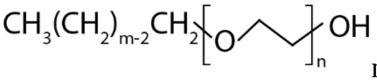 m = 14 n = 6	478.703	0.0063-0.01 [2,4,5]	0.9984 [4]
C12E5	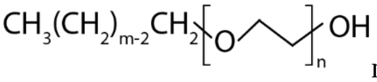 m = 12 n = 5	406.597	0.063-0.07 [2,4,6]	0.963†
C10E8	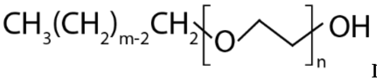 m = 10 n = 8	510.702	0.97-1.5 [2,4,7]	0.9987 [4]
Tween20	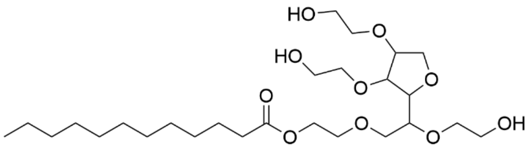	1227.53	0.06-0.08 [4,8,9]	1.1†
OTG	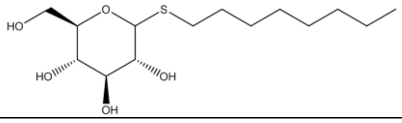	308.43	8.5-9.2 [4,10,11]	0.9998 [4]

† Stated on bottle from chemical supplier.

Table S2: Minimum surface tensions (mN/m) for ternary surfactant systems used to constrain droplet surface tension models.

	0.5 M NaCl	0.9 M glutaric acid
C16E8	37	38
C14E6	32	35
C12E5	30	33
C10E8	36	38
Tween20	34	37
OTG	30	34

Table S3: Average total concentration (mM) required to reach the minimum surface tension in 6 – 9 μm radius droplets. The total surfactant concentration to reach the droplet bulk was estimated as the concentration where the surface tension plateaus.

0.5 M NaCl					
	Monolayer	Simple Kinetic	Droplet Measurement Estimate	Monolayer/ Droplet Estimate	Simple Kinetic/ Droplet Estimate
C16E8	1.33	2.87	1.85	0.719	1.55
C14E6	2.05	1.20	2.60	0.788	0.462
C12E5	2.22	1.52	2.20	1.01	0.691
C10E8	1.99	1.57	2.00	0.995	0.785
Tween20	1.18	1.95	2.20	0.536	0.886
OTG	6.16	7.95	3.50	1.76	2.27
0.9 M Glutaric acid					
	Monolayer	Simple Kinetic	Droplet Measurement Estimate	Monolayer/ Droplet Estimate	Simple Kinetic/ Droplet Estimate
C16E8	1.25	1.89	1.50	0.833	1.26
C14E6	1.47	0.91	1.75	0.840	0.520
C12E5	1.70	1.30	1.90	0.895	0.684
C10E8	2.36	2.75	1.75	1.35	1.57
Tween20	1.01	1.19	1.85	0.546	0.643
OTG	26.19	21.11	13.00	2.015	1.624

Table S4: Integrated area (mM·mN/m) between 6 and 9 µm radius predictions for the Monolayer and Simple Kinetic Models for ternary surfactant systems.

0.5M NaCl			
	Monolayer	Simple Kinetic	Monolayer/ Simple Kinetic
C16E8	302	626	0.482
C14E6	681	712	0.956
C12E5	727	746	0.975
C10E8	250	595	0.420
Tween20	624	675	0.924
OTG	229	473	0.484
0.9 M Glutaric Acid			
	Monolayer	Simple Kinetic	Monolayer/ Simple Kinetic
C16E8	403	416	0.969
C14E6	474	476	0.996
C12E5	487	486	1.00
C10E8	199	319	0.624
Tween20	426	436	0.977
OTG	18	111	185.36

Table S5: MAE (mN/m) for Monolayer and Simple Kinetic Models 0.5 M NaCl or 0.9 M glutaric acid and surfactant mixtures and MASE model comparison.

	0.5 M NaCl			0.9 M Glutaric Acid		
	Monolayer MAE	Simple Kinetic MAE	MASE	Monolayer MAE	Simple Kinetic MAE	MASE
C16E8	1.84	11.70	0.16	1.77	2.32	0.77
C14E6	5.25	1.07	4.88	1.74	1.08	1.61
C12E5	4.39	1.82	2.41	2.67	1.70	1.34
C10E8	5.50	3.01	1.83	3.56	3.70	0.96
Tween20	4.37	9.01	0.49	1.22	3.35	0.37
OTG	3.81	6.28	0.61	5.98	5.40	1.11

SI Text 1: Monolayer Model Maximum Surface Excess Calculation

The total number of moles of surfactant in the droplet can be calculated from the total surfactant concentration (C_s) and the volume of the droplet which depends on its radius (r). The total surfactant moles are equal to the sum of the moles at the surface (n_s) and in the droplet bulk (n_b). The ratio of moles at the droplet surface to those in the bulk is a known quantity from the model output $\left(x = \frac{n_s}{n_b}\right)$. Using this ratio and the total number of moles of surfactant, it is possible to calculate the number of moles at the droplet surface. We then calculate the surface area (SA) of the droplet. Finally, the surface concentration is the calculated from the moles at the droplet surface and the droplet surface area.

$$n_t = C_s \frac{4\pi r^3}{3}$$

$$n_t = n_b + n_s = \frac{n_s}{x} + n_s$$

$$n_s = \frac{n_t}{\frac{1}{x} + 1}$$

$$SA = 4\pi r^2$$

$$\text{surface concentration} = \frac{n_s}{SA}$$

References

(1) Rusdi, M.; Moroi, Y.; Hlaing, T.; Matsuoka, K. Micelle Formation and Surface Adsorption of Octaethylene Glycol Monoalkyl Ether (CnE8). *Bull. Chem. Soc. Jpn.* **2005**, *78*, 604–610.

(2) Mattei, M.; Kontogeorgis, G. M. Modeling of the Critical Micelle Concentration (CMC) of Nonionic Surfactants with an Extended Group-Contribution Method. *Ind. Eng. Chem. Res.* **2013**, *52*, 12236–12246.

(3) Yan, C.; Angus-Smyth, A.; Bain, C. D. Adsorption Kinetics of Non-Ionic Surfactants in Micellar Solutions: Effects of Added Charge. *Faraday Discuss.* **2013**, *160*, 45–61. https://doi.org/10.1039/c2fd20118f.

(4) Bain, A.; Ghosh, K.; Prisle, N. L.; Bzdek, B. R. Surface-Area-to-Volume Ratio Determines Surface Tensions in Microscopic, Surfactant-Containing Droplets. *ACS Cent. Sci.* **2023**, *9*, 2076–2083. https://doi.org/10.1021/acscentsci.3c00998.

(5) Theander, K.; Pugh, R. J. Synergism and Foaming Properties in Mixed Nonionic/Fatty Acid Soap Surfactant Systems. *J. Colloid Interface Sci.* **2003**, *267* (1), 9–17. https://doi.org/10.1016/S0021-9797(03)00482-X.

(6) Binks, B. P.; Fletcher, P. D. I.; Paunov, V. N.; Segal, D. Equilibrium and Dynamic Adsorption of C12E5 at the Air-Water Surface Investigated Using Ellipsometry and Tensiometry. *Langmuir* **2000**, *16* (23), 8926–8931. https://doi.org/10.1021/la000371t.

(7) Bakshi, M. S.; Kaura, A.; Mahajan, R. K. Effect of Temperature on the Micellar Properties of Polyoxyethylene Glycol Ethers and Twin Tail Alkylammonium Surfactants. *Colloids Surfaces A Physicochem. Eng. Asp.* **2005**, *262* (1–3), 168–174. https://doi.org/10.1016/j.colsurfa.2005.04.027.

(8) Shah, V.; Bharatiya, B.; Shukla, A. D.; Mukherjee, T.; Shah, D. O. Adsorption of Nonionic Brij and Tween Surfactants at PTFE-Water and Air-Water Interfaces: Investigations on Wetting, Dispersion Stability, Foaming and Drug Solubilization. *Colloids Surfaces A Physicochem. Eng. Asp.* **2016**, *508*, 159–166. https://doi.org/10.1016/j.colsurfa.2016.08.057.

(9) Tran, C. D.; Yu, S. Near-Infrared Spectroscopic Method for the Sensitive and Direct Determination of Aggregations of Surfactants in Various Media. *J. Colloid Interface Sci.* **2005**, *283* (2), 613–618. https://doi.org/10.1016/j.jcis.2004.09.031.

(10) Molina-Bolívar, J. A.; Aguiar, J.; Peula-García, J. M.; Ruiz, C. C. Surface Activity, Micelle Formation, and Growth of n-Octyl-β-D-Thioglucopyranoside in Aqueous Solutions at Different Temperatures. *J. Phys. Chem. B* **2004**, *108* (34), 12813–12820. https://doi.org/10.1021/jp0480551.

(11) Frindi, M.; Michels, B.; Zana, R. Ultrasonic Absorption Studies of Surfactant Exchange between Micelles and Bulk Phase in Aqueous Micellar Solutions of Nonionic Surfactants with a Short Alkyl Chain. 3. Surfactants with a Sugar Head Group. *J. Phys. Chem.* **1992**, *96* (20), 8137–8141. https://doi.org/10.1021/j100193a082.
